# Optimizing automated white matter hyperintensity segmentation in individuals with stroke

**DOI:** 10.3389/fnimg.2023.1099301

**Published:** 2023-03-09

**Authors:** Jennifer K. Ferris, Bethany P. Tavenner, Mohamed Salah Khlif, Amy Brodtmann, Lara A. Boyd, Sook-Lei Liew

**Affiliations:** ^1^Graduate Program in Rehabilitation Sciences, University of British Columbia, Vancouver, BC, Canada; ^2^Gerontology Research Centre, Simon Fraser University, Vancouver, BC, Canada; ^3^Chan Division of Occupational Science and Occupational Therapy, University of Southern California, Los Angeles, CA, United States; ^4^Cognitive Health Initiative, Central Clinical School, Monash University, Melbourne, VIC, Australia; ^5^Department of Medicine, Royal Melbourne Hospital, Melbourne, VIC, Australia; ^6^Department of Physical Therapy, Faculty of Medicine, University of British Columbia, Vancouver, BC, Canada; ^7^Department of Neurology, Stevens Neuroimaging and Informatics Institute, Keck School of Medicine, University of Southern California, Los Angeles, CA, United States

**Keywords:** white matter hyperintensity (WMH), stroke, lesion segmentation, SAMSEG, FSL, BIANCA

## Abstract

White matter hyperintensities (WMHs) are a risk factor for stroke. Consequently, many individuals who suffer a stroke have comorbid WMHs. The impact of WMHs on stroke recovery is an active area of research. Automated WMH segmentation methods are often employed as they require minimal user input and reduce risk of rater bias; however, these automated methods have not been specifically validated for use in individuals with stroke. Here, we present methodological validation of automated WMH segmentation methods in individuals with stroke. We first optimized parameters for FSL's publicly available WMH segmentation software BIANCA in two independent (multi-site) datasets. Our optimized BIANCA protocol achieved good performance within each independent dataset, when the BIANCA model was trained and tested in the same dataset or trained on mixed-sample data. BIANCA segmentation failed when generalizing a trained model to a new testing dataset. We therefore contrasted BIANCA's performance with SAMSEG, an unsupervised WMH segmentation tool available through FreeSurfer. SAMSEG does not require prior WMH masks for model training and was more robust to handling multi-site data. However, SAMSEG performance was slightly lower than BIANCA when data from a single site were tested. This manuscript will serve as a guide for the development and utilization of WMH analysis pipelines for individuals with stroke.

## 1. Introduction

White matter hyperintensities (WMHs) are a form of cerebral small vessel disease that occur with aging and are associated with cardiometabolic risk factors (Jeerakathil et al., 2004; Launer, 2004). WMHs are also a significant risk factor for stroke; individuals with high WMH volumes are three time more likely to experience a stroke after adjustment for vascular risk factors (Debette and Markus, 2010). Consequently, WMHs are common in individuals with stroke (Wen and Sachdev, 2004), and WMHs may impact recovery outcomes after stroke (Helenius and Henninger, 2015; Georgakis et al., 2019). WMHs are fairly predictable in shape and distribution, making them excellent candidates for automated lesion segmentation pipelines (Balakrishnan et al., 2021). However, stroke lesions are highly variable in shape, size, and distribution (Bonkhoff et al., 2021), and often present challenges to automated MRI tools (Ito et al., 2019). Thus, automated tools for segmenting WMHs should be specifically validated for use in individuals with stroke.

Brain Intensity AbNormality Classification Algorithm (BIANCA) is an automated WMH segmentation software freely available from FSL (Griffanti et al., 2016). BIANCA employs supervised learning using a *k*-nearest neighbors (*k*-NN) algorithm (Griffanti et al., 2016). BIANCA has shown good segmentation accuracy across a variety of studies in older adults (Griffanti et al., 2016; Vanderbecq et al., 2020; Hotz et al., 2022), and requires relatively small amounts of training data in order to achieve good performance (Griffanti et al., 2016). BIANCA is now the WMH segmentation method of choice for many large-scale neuroimaging studies such as UK Biobank (Alfaro-Almagro et al., 2018). For stroke researchers, BIANCA is an appealing tool for WMH segmentation because it is publicly available and has established use in aging populations. However, MRI analytic tools developed in the aging brain may or may not generalize for use after stroke, and BIANCA has not been specifically validated for use in individuals with overt stroke lesions. The first aim of this manuscript is to determine the optimal analysis protocol to minimize potential effects of stroke lesions on WMH segmentation with BIANCA.

The second aim of this manuscript is to provide recommendations for the choice of segmentation method for stroke researchers, depending on the composition of their study cohort. In response to the need for larger sample sizes to adequately power neuroimaging studies of stroke recovery, the Enhancing Neuroimaging Genetics through Meta-Analysis (ENIGMA) Stroke Recovery working group is collating large datasets of individuals with stroke from multiple sites across the world (Liew et al., 2022a). This approach allows for the re-use of previously collected MRI scans and enhances the potential for novel discoveries. However, as a supervised learning method, BIANCA's performance may decrease when segmenting data that is different from the training dataset. Therefore, the use of BIANCA for multi-site data where MRI scanner or acquisition parameters differ from those of the training sample may be limited, though this has not been widely explored. In this study, we validated our optimized BIANCA protocol across two independent samples of individuals with stroke with different MRI acquisition parameters. We compared BIANCA performance when the model was trained and tested within the same dataset, when the model was trained on data from one dataset and tested on an independent dataset, and when the model was trained and tested on mixed data from both samples. We also evaluated the performance of an automated WMH segmentation with Sequence Adaptive Multimodal SEGmentation (SAMSEG), which is a contrast-based method that is unsupervised and expected to perform well on multisite data (Cerri et al., 2021). SAMSEG is freely available through FreeSurfer (version 7.2) (Puonti et al., 2016; Cerri et al., 2021) and is fully automated, meaning it does not have user-defined parameters that require optimization. SAMSEG performs lesion segmentation in the context of whole brain modeling, incorporating both T1- and T2-weighted images as inputs. SAMSEG employs unsupervised Gaussian mixture modeling to automatically group together voxels with similar intensities and perform voxel segmentation. SAMSEG learns appropriate intensity cutoffs for each image, making it robust to between site and scanner differences (Puonti et al., 2016). Here, we compared these two automated segmentation methods and performed validation analyses on two independent stroke datasets.

## 2. Methods

### 2.1. Datasets

Data for this study were assembled from two research groups to optimize BIANCA parameters and test them on an independent sample. The following sections describe the imaging protocols and WMH segmentation procedures used for each of these datasets. A summary of participant demographics can be found in Table 1.

**Table 1:** Participant demographics.

	**Dataset 1: Chronic stroke cohort *n* = 43**	**Dataset 2: Subacute stroke cohort *n* = 120**
Age^a^	65 (9)	68 (12)
**Sex** ^b^		
F	13 (30%)	38 (32%)
M	30 (70%)	82 (68%)
Months post-stroke^a^	69 (59)	3 (1)

a Mean (SD);

b *n* (%).

#### 2.1.1. Dataset 1: Chronic stroke cohort for BIANCA protocol optimization and testing

The chronic stroke dataset was collected at the Brain Behavior Laboratory of the University of British Columbia (UBC). This dataset was comprised of 43 individuals with chronic stroke (>6 months post-stroke). Inclusion criteria were as follows: (1) age between 40 and 80 years old, (2) >6 months post first clinically diagnosed stroke, (3) no history of seizure/epilepsy, head trauma, a major psychiatric diagnosis, neurodegenerative disorders, or substance abuse. To optimize BIANCA parameters, 80% of this dataset (*n* = 34) was randomly selected for model training and cross validation. Once the optimized BIANCA parameters were determined they were tested on the remaining 20% of the dataset (*n* = 9).

MRI images were acquired at the UBC MRI Research Center on a 3.0T Phillips Achieva or Elition scanner (Philips Healthcare, Best, The Netherlands). We acquired the following structural scans: (^*^1^*^) a T1-weighted 3D magnetization-prepared rapid gradient-echo (MPRAGE) anatomical scan [repetition time (TR)/time to echo (TE)/inversion time (TI) = 3,000/3.7/905 ms, flip angle = 9°, voxel size = 1 mm isotropic, field of view (FOV) = 256 × 224 × 180 mm], (2) a fluid attenuated inversion recovery (FLAIR) scan (TR/TE/TI = 9,000/90/2,500 ms, flip angle = 90°, voxel size = 0.94 × 0.94 mm FOV = 240 × 191 × 144 mm, slice thickness = 3 mm), and (^*^3^*^) a combined T2-weighted (T2) and proton density (PD) scan (TR/TE1/TE2 = 2,500/9.5/90 ms, flip angle = 90°, voxel size = 0.94 × 0.94 mm, FOV = 240 × 191 × 144 mm, slice thickness = 3 mm).

Gold-standard WMH segmentation was performed with the Semi-Automated Brain Region Extraction (SABRE) Lesion Explorer pipeline, a semi-automated and validated pipeline (Ramirez et al., 2011, 2020). WMH masks were visually quality checked and false positive voxels were removed where necessary by a single experienced rater. Stroke lesions were manually drawn by a single experienced rater on co-registered FLAIR and T1 images. SABRE tools were used for skull stripping and intensity normalization of structural scans (Dade et al., 2004; Ramirez et al., 2020).

#### 2.1.2. Dataset 2: Subacute stroke cohort for independent validation of BIANCA protocol

The optimized BIANCA model was tested on an independent cohort of individuals with subacute stroke (3 months post-stroke) from the Cognition and Neocortical Volume after Stroke (CANVAS) Study (*n* = 120). Details of the full study protocol have previously been published (Brodtmann et al., 2014).

MRI images were acquired on a 3T Siemens Tim Trio scanner (Erlangen, Germany) at the Melbourne Brain Center, Austin Campus of the Florey Institute of Neuroscience and Mental Health. The following scans were acquired: (^*^1^*^) a T1-weighted 3DMPRAGE sequence anatomical scan (TR/TE/TI = 1,900/2.6/900 ms, flip angle = 9°, voxel size = 1 mm isotropic, FOV = 256 × 256 × 160 mm), (^*^2^*^) a FLAIR scan (TR/TE/TI = 6,000/380/2,100 ms, flip angle = 120°, voxel size 0.5 × 0.5 × 1 mm^3^, FOV = 512 × 512 × 160 mm).

Gold-standard WMH segmentation was performed with a semi-automated procedure. SAMSEG was used for initial seed WMH segmentation, and generated WMH masks were manually edited with custom MATLAB software. Stroke lesions were manually drawn by experienced raters on FLAIR images. Skull stripping and intensity normalization of structural scans was performed according to published ENIGMA protocols (Liew et al., 2022a,b).

### 2.2. BIANCA optimization

We optimized the BIANCA parameters on our training sample from Dataset 1 (*n* = 34 individuals for training). Model optimization was scored with leave-one-out cross validation and standard BIANCA scoring metrics (Griffanti et al., 2016). BIANCA was run in FSL v6.0.5.

BIANCA requires all scans have the same FoV and voxel dimensions. To use BIANCA across multi-site data with different acquisition parameters, we first registered scans to 1 mm MNI space. Because stroke lesions can cause distortions in non-linear registrations, we used linear registration to MNI space to avoid any stroke-lesion related warping in scan registrations (Liew et al., 2018).

#### 2.2.1. BIANCA overview

BIANCA uses a *k*-NN algorithm to classify voxels as WMH or non-WMH based on the nearest training data in feature space. The feature space in BIANCA captures information about voxel intensity and spatial characteristics; these features are extracted from the training set with labeled voxels (i.e.: voxel label as WMH or non-WMH from gold-standard WMH masks). BIANCA's output gives each voxel's probability of belonging to WMH or non-WMH class, based on the proportion of *k* neighbors belonging to that class. The final step in BIANCA is applying a threshold to the voxel probability distributions to assign each voxel to WMH or non-WMH classes. To determine the optimal BIANCA parameters in individuals with stroke, we: (^*^1^*^) tested the user-defined BIANCA settings available in the BIANCA toolkit, (^*^2^*^) adjusted the WMH thresholding using either a fixed or an adaptive thresholding approach, and (^*^3^*^) applied additional methods for handling stroke lesions to improve BIANCA accuracy.

BIANCA performance was rated using standard BIANCA scoring metrics (Griffanti et al., 2016). The calculated metrics compare gold-standard WMH masks to the BIANCA-derived WMH masks for each participant and evaluate the degree of overlap and volumetric correspondence between masks. We selected the Dice Similarity Index (SI), interclass correlation coefficient (ICC), and cluster-level false negative ratio (FNRc) as our key metrics of interest (Griffanti et al., 2016). SI and FNRc index degree of mask overlap, and ICC measures volumetric correspondence. ICC was computed as the agreement between the gold-standard and automatically generated WMH volumes, with the R package “irr.” We gave higher importance to FNRc over false-positive ratio, as we prioritized sensitivity to lesion detection. Decisions about optimal BIANCA settings were made based on the performance of these three metrics. In cases where key metrics did not agree, we chose the setting that gave better performance in 2/3 of these metrics.

#### 2.2.2. BIANCA settings

BIANCA has several user-defined options to optimize *k*-NN WMH segmentation [for a full description see: Griffanti et al. (2016)]. Briefly, these are:

A. The **MRI modalities** used as features in training data. In our dataset we always include T1 and FLAIR scans as training features. We tested the additional value of including T2-weighted scans as a training feature.
B. **Spatial weighting** of BIANCA by MNI coordinates. BIANCA can use MNI-registration coordinates to weight the probability of WMH classification, because WMHs occur more frequently in some regions (e.g., periventricular to lateral ventricles) than others (e.g., brainstem). Higher spatial weighting values increase a linear scaling factor that increases the probability weighting of MNI coordinates. We tested the following values: 0 (no spatial weighting), 1, 5, and 10.
C. **Patch size** to define the local average intensity for each MRI modality. A “patch” can be used for local averaging of MRI intensity around each voxel to improve the robustness of the segmentation to misregistration. A higher patch size increases the size of the kernel used for local averaging. We tested a 3D patch using the following values: 0 (no patch), 3, 6, and 9.
D. **Training point location** for non-WMH points. By default BIANCA selects non-WMH training points from any location in the brain except for those in the WMH mask (“any” location option). There are two additional options to constrain the selection of non-WMH training points: points that do not directly border the WMH mask (“noborder” option), or points that are directly bordering the WMH-mask (“surround” option). We tested each of these three non-WMH training point location settings.
E. The **number of training points** for both WMH and non-WMH training points. By default, BIANCA selects 2,000 training points at random in the WMH masks, and an equal number of non-WMH training points. The user can specify the number of WMH and non-WMH training points to use or can direct BIANCA to use all the points within the WMH mask and an equal number of non-WMH training points for each individual. We tested the following values: all WMH and equal non-WMH, 2,000 WMH and 2,000 non-WMH, and 2,000 WMH and 10,000 non-WMH points. During the optimization phases we tested further increasing the number of non-WMH training points (see Supplementary material). Because changing the number of training points also changes the probability threshold values, we tested 5 different thresholding options for each training point setting (0.8, 0.85, 0.9, 0.95, and 0.99)

To determine the optimal BIANCA model for use in individuals with stroke, we systematically tested each of these user-defined BIANCA settings on our training sample from Dataset 1. We used identical testing procedures as employed in Griffanti et al. (2016). We began by applying BIANCA with all default options, then varied each BIANCA setting while keeping all other settings constant, to isolate the effects of each setting on BIANCA performance. We tested a total of 27 different BIANCA setting configurations: MRI modalities (2 options), spatial weighting (4 options), patch size (4 options), training point location (3 options), and training point number (3 options + 5 thresholds each). We compared BIANCA performance in each configuration and selected each best-performing setting to be applied as the start point in subsequent testing phases. We then ran BIANCA with the determined best setting configuration and again systematically varied each of the BIANCA settings and re-scored performance to test if the optimal settings remained constant. Testing continued in these phases until optimal BIANCA settings were determined (i.e., the setting consistently provided the best scores on our training sample).

#### 2.2.3. BIANCA thresholding

We tested applying a fixed threshold to WMH probability maps vs. an adaptive threshold using LOCally Adaptive Threshold Estimation (LOCATE) (Sundaresan et al., 2019). LOCATE takes a lesion probability map based on distance from the cerebral ventricles as input, and provides spatially adaptive thresholding of the WMH segmentation, accounting for lesion load, shape, and location. LOCATE thresholding was performed on BIANCA output from optimized parameters for each subject, and performance was scored and compared to performance using the optimal fixed threshold determined from the previous testing round.

#### 2.2.4. Stroke-specific optimization steps

We tested additional settings around the handling of stroke lesions:

1. Removing the stroke lesion from the brain mask prior to BIANCA training was compared to removing the stroke lesion after BIANCA training. This allowed us to test masking the stroke lesion on BIANCA input vs. output. This step was performed before systematic BIANCA parameter optimization to determine the optimal starting point for BIANCA model testing.
2. Stroke mask dilation to mask the boundary of the stroke lesion. We anticipated possible false-positive WMH voxels at the boundaries of stroke lesions. We tested whether removal of these false positives might improve BIANCA performance by dilating the stroke lesion mask and removing the dilated mask from BIANCA output. We dilated the stroke lesion mask in 1 mm increments from 1 to 5 mm in size and scored BIANCA performance with the dilated masks removed.

#### 2.2.5. BIANCA model testing

Once the optimized BIANCA parameters were established in the training sample from Dataset 1 (chronic stroke cohort), we ran the optimized BIANCA model on the test sample from Dataset 1 cohort and scored performance. We then used the optimized BIANCA settings on Dataset 2 (subacute stroke cohort) in two phases: (1) by splitting Dataset 2 into an 80/20% training and test sample (*n* = 96 training; 24 testing) and applying BIANCA with the same testing parameters to confirm the optimized settings would transfer to a new cohort; (^*^2^*^) testing the BIANCA model trained on Dataset 1 to segment WMHs in Dataset 2. Finally, we evaluated BIANCA performance on combined data from both independent datasets, by training data on a mixed random sample of data from each dataset (*n* = 34 from Dataset 1 and 34 from Dataset 2) and testing the trained model on the remaining data (*n* = 9 from Dataset 1 and 86 from Dataset 2). These steps allowed us to evaluate the accuracy of applying a trained BIANCA model to a novel unseen dataset.

### 2.3. SAMSEG lesion segmentation

We further compared BIANCA output with SAMSEG lesion segmentation performance, with a particular interest in comparing performance on multi-site data. SAMSEG was run in Freesurfer v7.3.1. As mentioned previously, SAMSEG is an unsupervised and automated tissue segmentation method that uses parametric Bayesian modeling for tissue segmentations (Puonti et al., 2016; Cerri et al., 2021). WMH segmentation is implemented with additional unsupervised models to learn the shape and intensity of WMH lesions (Cerri et al., 2021). SAMSEG has excellent reliability across multi-site data (Puonti et al., 2016; Cerri et al., 2021). Because SAMSEG is an unsupervised method, it does not require the use of training WMH masks for tissue segmentation. We tested performance with different probability thresholds of voxels being assigned as lesion, with the following threshold values: 0.1, 0.3 (SAMSEG default setting), 0.5, 0.7, and 0.9.

T1 scans and FLAIR scans registered to T1 space were used as inputs to SAMSEG, and stroke lesion masks were used to remove stroke lesions from the resulting segmented SAMSEG tissue classes. SAMSEG was performed with the *run_samseg* command implemented through FreeSurfer (v.7.2). SAMSEG performance was scored against gold standard WMH masks for every individual.

## 3. Results

### 3.1. BIANCA optimization

**Figure 1:**
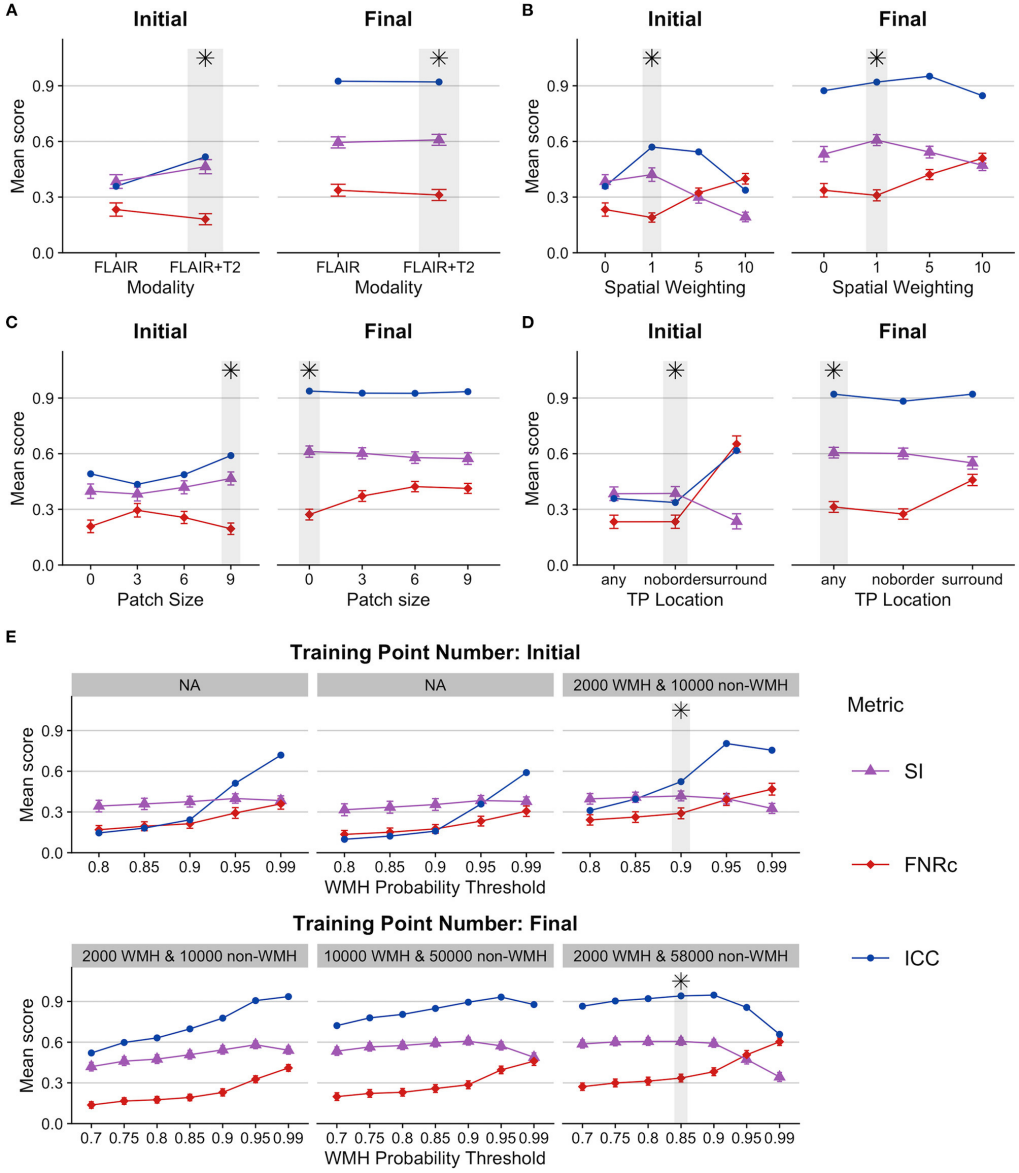
BIANCA parameter optimization. Figures present BIANCA model scoring for user-defined BIANCA settings tested in the initial and final rounds of BIANCA setting testing. BIANCA performance was scored against gold-standard WMH-masks with the dice similarity index (SI), false-negative ratio by cluster (FNRc), and interclass-correlation coefficient (ICC). Y-axis values are the mean scores for the corresponding scoring metrics. Gray bars and black asterisks indicate the best-performing setting for each BIANCA option. **(A–E)** BIANCA options, see methods “BIANCA Optimization” section for full description of each option [corresponding to tested options **(A–E)**]. For full performance scores from all rounds of BIANCA optimization, see Supplementary Tables 1–4.

Our data required four phases of systematic testing to determine the optimized user-defined BIANCA model settings. Results from BIANCA optimization for the first phase of setting testing and the final phase of setting testing are presented in Figure 1. The values of all BIANCA scoring measures across four phases of parameter testing are presented in Supplementary Tables 1–4.

General observations from parameter testing include the following:

1. Using more MRI modalities as features (T1, FLAIR, and T2 scans) always improved BIANCA performance.
2. Incorporating MNI coordinates with spatial weighting improved BIANCA performance. The best spatial weighting was 1.
3. Training point location performance was largely equivalent when the training points came from anywhere in the brain, or if they excluded the boundary around the WMH mask (“any” and “noborder” options). BIANCA performance decreased when training points were restricted to the WMH boundary (“surround” option).
4. BIANCA performance generally improved with higher numbers of non-WMH training points. Our final best performing model included 2,000 WMH and 58,000 non-WMH training points.

Our optimal BIANCA settings were consistent with the optimal settings determined in a non-stroke cohort by Griffanti et al. (2016), with one exception: we obtained better BIANCA performance with a higher number of non-WMH training points (58,000) than Griffanti et al. (2016) (10,000).

#### 3.1.1. Thresholding

**Figure 2:**
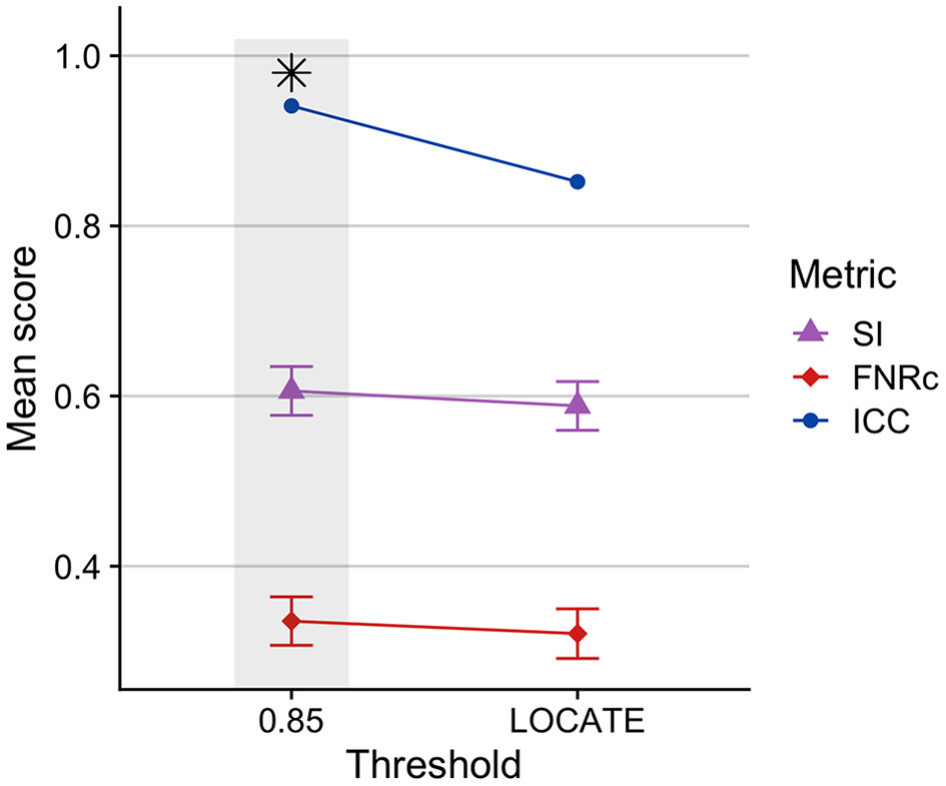
Comparison of BIANCA performance on Dataset 1 training sample using a fixed threshold (0.85) compared to an adaptive threshold using LOCATE. BIANCA performed better with a fixed threshold, indicated by higher SI and ICC values. A fixed threshold of 0.85 was used in the optimized BIANCA model. Gray bars and black asterisks indicate the best-performing setting.

Figure 2 presents a comparison between the optimized fixed threshold (0.85) and adaptive LOCATE-based threshold. We found the fixed threshold had better performance, with higher SI and ICC, though FNRc was also slightly higher with the fixed threshold.

#### 3.1.2. Stroke-specific optimization

**Figure 3:**
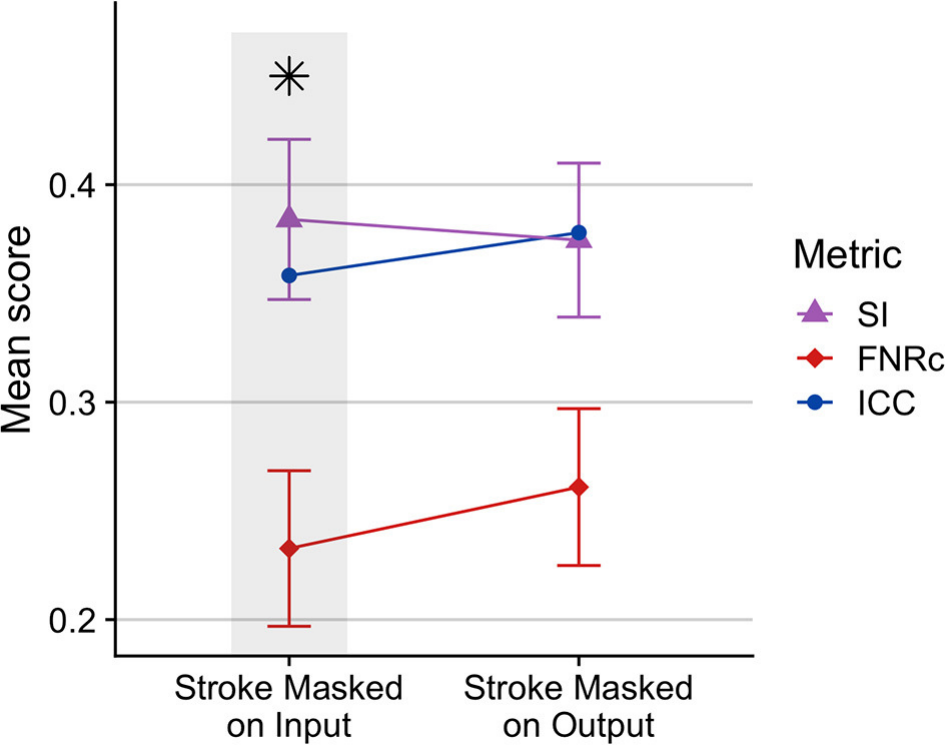
Comparison of BIANCA performance when stroke lesion was removed from BIANCA input or output. BIANCA performance was improved when stroke lesions were masked in training input. This testing phase was performed prior to BIANCA optimization to determine the best starting point for BIANCA model testing (default BIANCA parameters). Gray bars and black asterisks indicate the best-performing setting.

Stroke masking: Figure 3 compares excluding the stroke mask from BIANCA input vs. output. We found that WMH segmentation was improved when the stroke mask was excluded from model training on input. This was likely because the stroke lesion voxels were not included as non-WMH training points, thus improving WMH segmentation.

**Figure 4:**
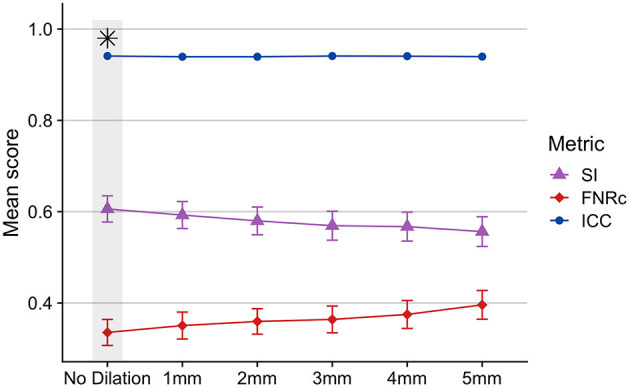
Effects of dilating the stroke lesion mask and removing from BIANCA output as a potential method to control for false positive WMHs around the boundaries of the stroke lesion. BIANCA performance decreased with increasing size of stroke lesion dilations, and the best BIANCA performance was achieved when stroke lesions were not altered in size. This indicates BIANCA did not identify significant numbers of false-positive WMHs around the boundaries of stroke lesions. This step was performed after BIANCA parameter optimization too fine-tune BIANCA model output (optimized BIANCA parameters). Gray bars and black asterisks indicate the best-performing setting.

Stroke lesion dilation: Figure 4 presents results of stroke lesion dilation on BIANCA performance scores. We found BIANCA performance was best when the stroke mask was not dilated in size, and performance decreased with increased stroke lesion dilation sizes.

#### 3.1.3. Optimized BIANCA model summary

**Figure 5:**
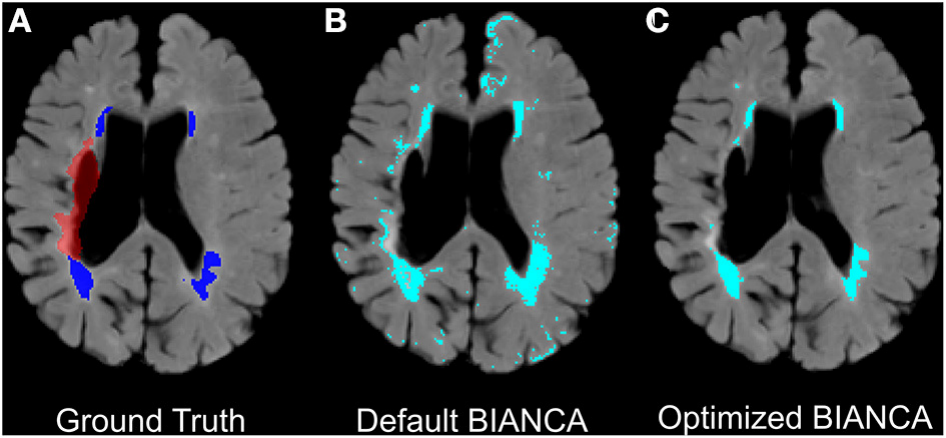
Example improvements in BIANCA WMH segmentation in an individual with chronic stroke. Panels present **(A)** ground truth WMH masks from SABRE segmentation (in dark blue) and the stroke lesion mask (in red-excluded from BIANCA segmentations), **(B)** + **(C)**: automated WMH segmentation between default BIANCA options **(B)** and BIANCA segmentation with the set of optimized BIANCA parameters described in the current report **(C)**.

Our final optimized BIANCA model had the following parameters: (^*^1^*^) stroke lesion masking on data input, (^*^2^*^) FLAIR, T1 and T2 scans included as training modalities, (^*^3^*^) MNI coordinates incorporated with a SW = 1, (^*^4^*^) training point location anywhere, with 2,000 WMH training points and 58,000 non-WMH training points, and (^*^5^*^) a threshold of 0.85 applied to BIANCA output. These BIANCA settings resulted in good performance on the training sample with the following performance scores: SI = 0.61, ICC = 0.94, FNRc = 0.34 (Table 2). WMH segmentation was greatly improved with optimized BIANCA settings when compared to the default BIANCA settings (Figure 5).

**Table 2:** WMH segmentation performance scores.

**Dataset**	**Training data**	**Overlap with gold-standard mask**						**Volumetric correspondence**	
		**SI**	**FDR**	**FNR**	**FDRc**	**FNRc**	**DER**	**OER**	**ICC**
**BIANCA**									
Dataset 1	Dataset 1	0.60	0.35	0.36	0.60	0.35	0.12	0.67	0.93
Dataset 2	Dataset 2	0.65	0.30	0.37	0.51	0.55	0.13	0.58	0.95
Dataset 2	Dataset 1	0.08	0.95	0.14	0.97	0.13	0.09	1.75	0.01
Mixed sample	Mixed sample	0.54	0.47	0.41	0.79	0.55	0.24	0.69	0.95
**SAMSEG**									
Dataset 1		0.54	0.57	0.16	0.77	0.34	0.20	0.73	0.95
Dataset 2		0.54	0.33	0.49	0.53	0.76	0.45	0.48	0.95

Table presents performance scores for Dataset 1 (chronic stroke cohort) and Dataset 2 (subacute stroke cohort), comparing performance between WMH segmentation methods (BIANCA vs. SAMSEG) and datasets used in supervised model training (BIANCA). SI, Dice similarity index; FDR, false-discovery ratio; FNR, false-negative ratio; FDRc, cluster-level FDR; FNRc, cluster-level FNR; DER, detection error rate; OER, outline error rate; ICC, interclass correlation coefficient.

### 3.2. BIANCA validation

#### 3.2.1. Testing dataset

Our optimized BIANCA parameters were tested on the reserved 20% of our training sample (*n* = 9). BIANCA achieved good performance on the test data set with the following performance scores: SI = 0.60, ICC = 0.91, FNRc = 0.42 (Table 2).

#### 3.2.2. Independent cohort validation

We validated BIANCA performance in an independent cohort of scans from a subacute stroke population (Dataset 2). First, we validated the optimized BIANCA settings by training and testing BIANCA on data from Dataset 2. Using the optimized BIANCA parameters gave good performance on the training and test sample from Dataset 2 (training sample: SI = 0.60, ICC = 0.95, FNRc = 0.54; test sample: SI = 0.66, ICC = 0.96, FNRc = 0.55). Next, we tested if the BIANCA model trained on Dataset 1 could be applied to segment WMHs in Dataset 2. BIANCA had very poor performance when the model trained off data from Dataset 1 was applied to segment WMHs in Dataset 2, with the following performance scores: SI = 0.08, ICC = 0.01, FNRc = 0.13. Finally, we tested BIANCA performance when trained and tested off a mixed sample of data from Datasets 1 and 2. BIANCA maintained good performance when trained off mixed sample data, with the following performance scores: SI = 0.54, ICC = 0.95, FNR_c_ = 0.55 (Table 2).

### 3.3. SAMSEG segmentation

We compared SAMSEG performance on multisite data. SAMSEG is an unsupervised segmentation method, therefore no training data are needed for WMH segmentation. SAMSEG performance was best with a threshold of 0.1 (Supplementary Figure 1, Supplementary Table 5), this threshold setting was subsequently applied to all SAMSEG output.

**Figure 6:**
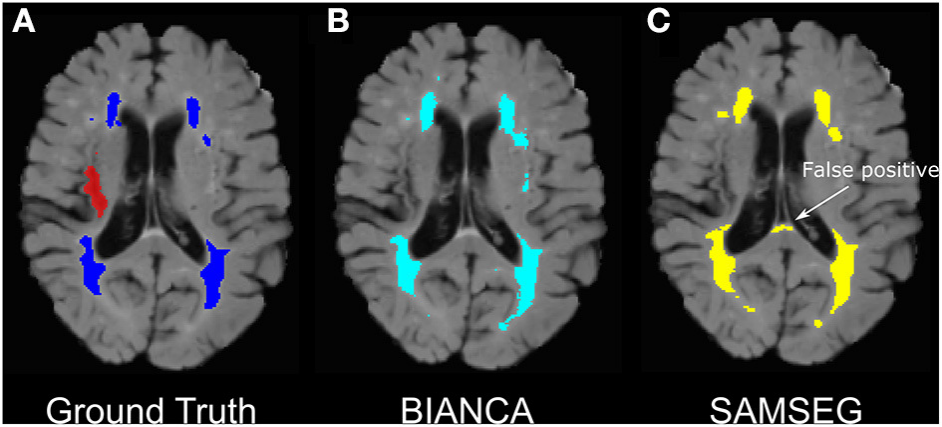
Example of false-positive WMH segmentation errors in BIANCA vs. SAMSEG in an individual with chronic stroke. **(A)** Ground truth WMH masks from SABRE segmentation (in dark blue) and the stroke lesion mask (in red- excluded from WMH segmentations), **(B)** BIANCA WMH segmentation. **(C)** SAMSEG false positives segmenting portions on the corpus callosum as WMHs.

SAMSEG achieved good performance on Dataset 1 (SI = 0.54, ICC = 0.95, FNRc = 0.34). On Dataset 2 SAMSEG also achieved good performance, however the false negative ratio was high (SI = 0.54, ICC = 0.95, FNRc = 0.76). Generally, SI scores were lower with SAMSEG compared to BIANCA, but ICC scores were comparable between the two methods. Outcome metrics from all tested BIANCA and SAMSEG models are presented in Table 2. Qualitatively, we noticed that SAMSEG had a greater chance of false positive in the corpus callosum (Figure 6).

### 3.4. WMH segmentation overview

**Figure 7:**
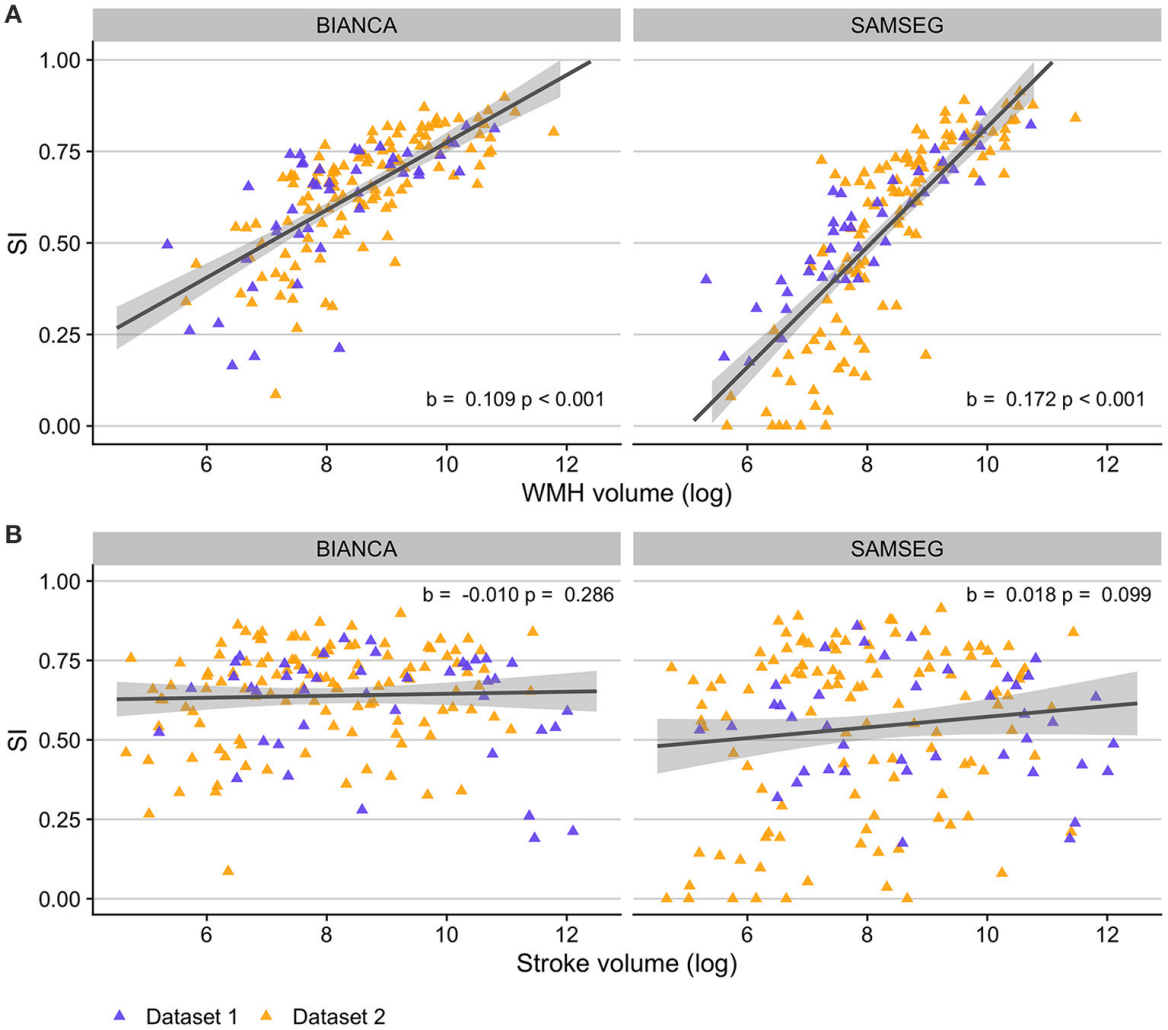
Relationships between automated WMH segmentation performance (indexed by Dice similarity index; SI) and lesion volumes in individuals with stroke. **(A)** Individuals with higher WMH volumes had better automated WMH segmentation accuracy with both BIANCA (left) and SAMSEG (right) segmentation algorithms. **(B)** WMH segmentation accuracy did not relate to stroke volumes for either BIANCA or SAMSEG segmentation. Dataset 1: cohort of individuals with chronic stroke; Dataset 2: cohort of individuals with subacute stroke.

Figure 7 presents relationships between BIANCA and SAMSEG performance and lesion volumes. After controlling for age and time post-stroke, there was a linear relationship between log-transformed WMH volumes and SI scores for both BIANCA (*b* = 0.109, *p* < 0.001) and SAMSEG (*b* = 0.172, *p* < 0.001) performance. There was no relationship between log-transformed stroke volumes and BIANCA (*b* = −0.010, *p* = 0.286) or SAMSEG (*b* = 0.018, *p* = 0.099) performance.

## 4. Discussion

In this manuscript we developed a set of optimized parameters for WMH segmentation with BIANCA in individuals with stroke. Our optimized BIANCA protocol demonstrated good performance on both a chronic stroke and a subacute stroke cohort when trained and tested within the same cohort. As a supervised learning technique, BIANCA's performance failed when tested on data with different acquisition parameters from the training data, but good performance was maintained if BIANCA was trained off mixed sample data from two independent datasets. We also tested the performance of FreeSurfer's unsupervised contrast-based WMH segmentation tool SAMSEG. Compared to BIANCA, SAMSEG had slightly poorer Dice similarity index scores and higher false negative ratios, but still gave good WMH segmentation performance in individuals with stroke. Importantly, SAMSEG maintained good performance scores across multi-site data without the need for model training and therefore may be a more practical method for use in large multi-center research studies. A comparison of each technique is presented in Table 3.

**Table 3:** Comparison of BIANCA and SAMSEG for use in individuals with stroke.

	**Pros**	**Cons**
BIANCA	- Better segmentation performance, particularly in spatial performance	- Poor performance when generalizing trained model to multi-site data
	- Better detection of deep WMHS	- Stroke lesion masking is critical
		- High quality WMH masks need for model training
SAMSEG	- Quick and easy segmentation package, fully automated	- Spatial performance worse than BIANCA
	- Can be applied across multi-site data	- Higher rate of false positive and false negative WMHs

### 4.1. BIANCA

BIANCA is a publicly available software tool that is easily implemented and widely used for WMH segmentation (Griffanti et al., 2016). While there have been some efforts to develop machine learning-based methods specifically for stroke and WMH segmentation in stroke populations (Guerrero et al., 2018) these are yet to be openly available and widely implemented. For now, the best choice for the stroke research field is to adapt automated methods developed in otherwise healthy individuals for use in individuals with stroke. We found that BIANCA performs well for WMH segmentation in individuals with stroke using the optimized set of parameters described here.

Recommendations for BIANCA in individuals with stroke:

1. The stroke lesion should be excluded from the input training data by removing the stroke lesion from the brain mask.
2. Including multiple imaging modalities (FLAIR, T1, and T2 scans) improves BIANCA performance, if available.
3. Registration of input scans to MNI space is recommended; BIANCA performance was always best when MNI coordinates were incorporated with a spatial weighting of 1.
4. The number of non-WMH training points should be increased beyond default settings (we recommend 2,000 WMH points and 58,000 non-WMH points).
5. We recommend use of a fixed threshold (0.85 in the current report) rather than an adaptive threshold using LOCATE to binarize generated BIANCA probability maps.

In our study BIANCA's similarity index scores were lower than what has been achieved with BIANCA in typical aging (Sundaresan et al., 2019). However, our similarity index scores were similar to scores in individuals with mild cerebrovascular disease such as transient ischemic attack (Griffanti et al., 2016), and were in line with typical similarity index scores for automated stroke lesion segmentation (Ito et al., 2019). Thus, our observed spatial performance was within typical similarity index scores achieved for individuals with stroke, where increased variability in brain structure is expected to impact the performance of automated MRI tools (Ito et al., 2019). Additionally, our observed ICC scores were excellent, indicating good volumetric correspondence in BIANCA WMH segmentation.

Generally, BIANCA was able to handle the presence of a stroke infarct without significant additional processing steps. BIANCA performed best when stroke lesions were excluded from the training data, because voxels containing abnormal signal from stroke lesions were not used as training points in the BIANCA algorithm. This principle may hold true for supervised brain tissue segmentation for other major neurological pathologies in clinical populations. We did not see any benefit (and in fact performance decreased) when the stroke lesion mask was dilated in size to avoid including voxels around the lesion in the data.

Using multiple imaging modalities beyond FLAIR as training features improved performance; this has been a consistent finding in the literature (Griffanti et al., 2016; Ling et al., 2018). We did not test the inclusion of additional structural scans that are routinely collected in stroke research studies (such as DWI or proton density scans), and BIANCA performance might be further improved through inclusion of additional MRI modalities as training features. However, both BIANCA and SAMSEG maintained good performance when using only one T2-weighted modality, as evidenced by results from our optimization testing for Dataset 1 (comparing the inclusion of FLAIR vs. FLAIR and T2 scans) and all results for Dataset 2 (which only had FLAIR and T1 scans acquired). WMH segmentation was also improved through the incorporation of MNI coordinates through a linear MNI registration. Stroke lesions can induce distortions in non-linear registrations, which requires careful analytic approaches to overcome such as careful lesion masking or enantiomorphic normalization of lesioned tissue (Nachev et al., 2008; Ito et al., 2018). We used linear registration to bring images into a common space without registration distortions from stroke lesions. This improved WMH segmentation after incorporating MNI coordinates through spatial weighting. An important consideration with this approach is that linear registrations bring a trade-off such that the specific concurrence with atlas-based region definitions will be reduced relative to what can be achieved with high-quality non-linear registrations. Furthermore, because our study only used linear registration, we were unable to test the additional benefit of regional masking procedures to reduce false positives implemented in BIANCA through mask_brain_mask, because this step requires non-linear registration warps to run. If high-quality non-linear registrations are available within a stroke cohort, then this additional step could be taken to constrain the BIANCA training space and potentially further improve WMH segmentation.

The tool LOCATE uses a spatially-adaptive technique to threshold BIANCA WMH probability maps (Sundaresan et al., 2019). We found the adaptive LOCATE threshold resulted in worse performance when compared to a fixed threshold, in contrast to what has been reported in older adults (Sundaresan et al., 2019). This might be because of increased neurological variability in individuals with stroke from the stroke lesion itself and concurrent age-related neurodegeneration and cerebral atrophy (Wen and Sachdev, 2004; Duering et al., 2012; Brodtmann et al., 2020). This increased neurological variability may make it more difficult for the adaptive threshold process to identify the optimal thresholds to apply across the brain. The use of a fixed threshold has additional benefits beyond improved accuracy, as a fixed threshold is simpler to implement and requires less computational time in the processing pipeline.

BIANCA similarity index performance was linearly related to WMH volumes. Smaller WMH volumes were more difficult to accurately segment, this has also been reported elsewhere with BIANCA (Wulms et al., 2022) and other automated WMH segmentation algorithms (Heinen et al., 2019). For small WMH volumes, a spatial disagreement of only a few voxels can have a large impact on spatial performance measures. Importantly, we found no relationship between similarity index scores and total stroke volume. This means that BIANCA was able to accurately segment WMHs even in individuals with large stroke lesions and is a robust technique to use in individuals with stroke.

As a supervised method, BIANCA did not perform well when tested on data with different acquisition parameters from the training data. This has implications for the use of BIANCA for large multi-site studies. If training WMH masks are available from each site, then good BIANCA performance can be achieve with site-specific or mixed-site training of the algorithm, a finding that has also been observed in samples of older adults (Bordin et al., 2021). If training WMH masks are not available for each site, and the goal of the study is to harmonize data across multiple sites with different acquisition parameters, then BIANCA is not the optimal WMH segmentation method.

BIANCA relies on a k-NN algorithm, which is one of the most commonly used algorithms applied to date for supervised WMH segmentation (Frey et al., 2019). Many of the findings of our study would generalize to the use any supervised WMH segmentation algorithm, for instance in the need to mask out stroke lesions from input to the training data. Recent advancements in deep learning methods, particularly convolutional neural networks, have shown excellent preliminary results for WMH segmentation in older adults (Kuijf et al., 2019; Isensee et al., 2021). Many of these algorithms are not yet publicly available, and their accuracy in individuals with concurrent stroke lesions remains to be established. However, the development of more advanced machine learning models has high potential to further improve automated segmentation of both WMH and stroke lesions in the future.

### 4.2. SAMSEG

SAMSEG was recently developed for segmentation of multiple sclerosis (MS) lesions (Cerri et al., 2021), and it also has been used to segment age-related WMHs (Restrepo et al., 2021; Dewenter et al., 2022). Unlike BIANCA, SAMSEG does not require tuning of parameters for each sample, nor does it require WMH masks for model training, making it a quick and practical tool for segmentation of large datasets. SAMSEG performance was comparable across data from two different research sites without the need for model training to site-specific sequences.

Despite achieving excellent volumetric correspondence scores (ICC) and good similarity index scores, SAMSEG had a relatively high rates of cluster-level false positives with our applied threshold of 0.1, with false-positive lesion frequently appearing in the midline of corpus callosum white matter. Our study prioritized sensitivity to lesion detection (low false negatives) over false positives, and our low probability threshold of 0.1 achieved this balance. However, depending on the goals of the research study, a higher probability threshold could be applied which would decrease the rate of false positives, but increase false negatives. False negatives may be most likely for small deep WMHs (WMHs that do not contact the cerebral ventricles). Deep WMHs are notoriously difficult to segment with automated methods (Park et al., 2018). Furthermore, WMH segmentation tools that were developed for MS lesion segmentation can show decreased performance on age-related WMHs due to reduced gray matter/white matter contrast in the aging brain (Caligiuri et al., 2015). Therefore, analytic choices can be made weighting the sensitivity to lesion detection and spatial accuracy vs. wholistic volumetric correspondence, depending on the aims of the analysis.

SAMSEG performance was not impacted by stroke lesion volumes, meaning SAMSEG performed equally well in individuals with large and small stroke lesions. SAMSEG is publicly available through FreeSurfer's platform, but SAMSEG can be run independently from the full FreeSurfer processing pipeline with coarser regional parcellation. While FreeSurfer frequently fails in the presence of stroke lesions (Ozzoude et al., 2020), SAMSEG did not show any failures or decrement in WMH segmentation performance in individuals with stroke. Additionally SAMSEG does not require any preprocessing steps (Puonti et al., 2016), meaning there are no steps to handle the stroke lesion on data input. Here, we removed stroke masks from output SAMSEG tissue segmentations as a post-processing step. If SAMSEG is applied in a cohort where stroke lesion masks are not available for this post-processing step, we recommend checking segmentation output carefully for any misclassification of stroke lesion tissue as a WMH. Additionally, we did not evaluate the accuracy of other tissue segmentations (gray matter, white matter, CSF) from SAMSEG output in individuals with stroke.

A final limitation for researchers to consider when choosing an automated WMH segmentation method is that both BIANCA and SAMSEG rely on high quality structural scans, such as those generated on research MRI scanners. These methods may not generalize for use in clinically acquired scans, which typically have low through-plane resolution and often not amenable to MNI registration and 3D segmentation techniques. Clinical WMH segmentation methods may require specific WMH segmentation, such as those developed by the MRI-GENIE study (Schirmer et al., 2019).

## 5. Conclusions

In this paper, we present an optimized protocol for automated supervised WMH segmentation in individuals with stroke using BIANCA. We also compared BIANCA performance to SAMSEG, an unsupervised WMH segmentation method. Both BIANCA and SAMSEG achieved good WMH segmentation performance in the presence of stroke lesions. Our data validate the use of automated WMH segmentation methods in stroke research studies, with potential additional considerations for the handling of stroke lesions in the segmentation pipeline. With the acceleration of research examining the contributions of concurrent age-related cerebrovascular disease on stroke outcomes, we expect this paper to be a useful methodological guide for the selection of WMH segmentation technique depending on the study aims and data composition.

## Data availability statement

The original contributions presented in the study are included in the article/Supplementary material, further inquiries can be directed to the corresponding author.

## Ethics statement

The studies involving human participants were reviewed and approved by University of British Columbia Clinical Research Ethics Board, Austin Hospital Research Ethics Committee, Box Hill Hospital Research Ethics Committee, and Royal Melbourne Hospital Research Ethics Committee. The patients/participants provided their written informed consent to participate in this study.

## Author contributions

JF and S-LL conceived of and designed the study. MK, AB, and LB contributed data. JF and BT analyzed the data. JF wrote the manuscript. All authors contributed to editing the manuscript and approved the submitted version.
