# Supplementary material for: Optimizing automated white matter hyperintensity segmentation in individuals with stroke
Source: Front Neuroimaging. 2023 Mar 9;2:1099301. doi: 10.3389/fnimg.2023.1099301 (PMC10406248; doi:10.3389/fnimg.2023.1099301)
Supplement: Supplementary file 1 [file Data_Sheet_1.pdf]

Supplementary Material

Supplementary Table 1: First Round of BIANCA Settings Optimization
Model Starting Point: Default BIANCA Settings

BIANCA options		Overlap with manual mask						Volumetric correspondence	
Setting	Threshold	SI	FDR	FNR	FDRc	FNRc	DER	OER	ICC
Modality									
FLAIR	0.95	0.38	0.62	0.36	0.90	0.23	0.73	0.50	0.36
FLAIR+T2	0.95	0.46	0.60	0.27	0.90	0.18	0.55	0.52	0.52
Patch Size									
0	0.95	0.40	0.64	0.30	0.92	0.21	0.75	0.45	0.49
3	0.95	0.38	0.61	0.37	0.88	0.29	0.71	0.52	0.43
6	0.95	0.42	0.55	0.39	0.85	0.26	0.62	0.54	0.49
9	0.95	0.47	0.53	0.35	0.87	0.20	0.54	0.53	0.59
Spatial Weight									
0	0.95	0.38	0.62	0.36	0.90	0.23	0.73	0.50	0.36
1	0.95	0.42	0.66	0.23	0.93	0.19	0.36	0.80	0.57
10	0.95	0.19	0.86	0.48	0.94	0.40	0.36	1.26	0.34
5	0.95	0.30	0.78	0.35	0.94	0.32	0.46	0.94	0.54
Training Point Location									
any	0.95	0.38	0.62	0.36	0.90	0.23	0.73	0.50	0.36
noborder	0.95	0.39	0.62	0.34	0.90	0.23	0.73	0.50	0.34
surround	0.95	0.24	0.55	0.82	0.76	0.65	0.58	0.95	0.62
Training Point Number									
All WMH & equal non-WMH	0.8	0.34	0.71	0.22	0.94	0.17	0.87	0.45	0.15
	0.85	0.36	0.69	0.25	0.94	0.19	0.84	0.44	0.18
	0.9	0.38	0.66	0.29	0.93	0.21	0.81	0.44	0.24
	0.95	0.40	0.58	0.42	0.88	0.29	0.67	0.54	0.51
	0.99	0.38	0.53	0.55	0.85	0.36	0.56	0.68	0.72
2000 WMH & 2000 non-WMH	0.8	0.32	0.74	0.18	0.95	0.13	0.83	0.54	0.10
	0.85	0.33	0.72	0.20	0.94	0.15	0.83	0.50	0.12
	0.9	0.35	0.69	0.23	0.93	0.18	0.84	0.45	0.16
	0.95	0.38	0.62	0.36	0.90	0.23	0.73	0.50	0.36
	0.99	0.38	0.57	0.50	0.87	0.31	0.63	0.62	0.59
2000 WMH & 10000 non-WMH	0.8	0.40	0.62	0.31	0.90	0.24	0.74	0.47	0.31
	0.85	0.41	0.59	0.35	0.89	0.26	0.68	0.50	0.39
	0.9	0.42	0.56	0.41	0.87	0.29	0.62	0.54	0.52
	0.95	0.40	0.49	0.57	0.84	0.39	0.50	0.71	0.80
	0.99	0.33	0.47	0.71	0.80	0.47	0.44	0.91	0.76

SI: Dice similarity index; FDR: false-discovery ratio; FNR: false-negative ratio; FDRc: cluster-level FDR; FNRc: cluster-level FNR; DER: detection error rate; OER: outline error rate; ICC: interclass correlation coefficient

Supplementary Table 2: Second Round of BIANCA Settings Optimization
Model Starting Point: Modality = FLAIR + T2; Patch Size = 9; Spatial Weighting = 1; Training Point Location = noborder; Training Point Number = 2000 WMH & 10000 non-WMH; Threshold=0.9

BIANCA options		Overlap with manual mask						Volumetric correspondence	
Setting	Threshold	SI	FDR	FNR	FDRc	FNRc	DER	OER	ICC
Modality									
FLAIR	0.95	0.51	0.55	0.28	0.81	0.31	0.18	0.80	0.84
FLAIR+T2	0.95	0.52	0.58	0.23	0.78	0.26	0.18	0.79	0.75
Patch Size									
0	0.95	0.53	0.57	0.18	0.86	0.16	0.19	0.75	0.77
3	0.95	0.53	0.58	0.19	0.83	0.21	0.18	0.77	0.75
6	0.95	0.52	0.57	0.22	0.78	0.27	0.17	0.79	0.74
9	0.95	0.52	0.58	0.23	0.78	0.26	0.18	0.79	0.75
Spatial Weight									
0	0.95	0.52	0.52	0.30	0.81	0.22	0.40	0.56	0.70
1	0.95	0.52	0.58	0.23	0.78	0.26	0.18	0.79	0.75
10	0.95	0.38	0.70	0.35	0.76	0.46	0.20	1.05	0.82
5	0.95	0.45	0.65	0.28	0.73	0.41	0.17	0.94	0.82
Training Point Location									
any	0.95	0.52	0.56	0.25	0.79	0.29	0.19	0.76	0.79
noborder	0.95	0.52	0.58	0.23	0.78	0.26	0.18	0.79	0.75
surround	0.95	0.41	0.22	0.69	0.46	0.60	0.24	0.93	0.81
Training Point Number									
All WMH & equal non-WMH	0.8	0.26	0.83	0.12	0.95	0.13	0.38	1.10	0.27
	0.85	0.30	0.81	0.14	0.95	0.14	0.38	1.02	0.34
	0.9	0.35	0.76	0.16	0.93	0.18	0.40	0.90	0.43
	0.95	0.46	0.64	0.26	0.90	0.25	0.32	0.77	0.66
	0.99	0.51	0.55	0.35	0.86	0.32	0.26	0.73	0.83
2000 WMH & 2000 non-WMH	0.8	0.22	0.87	0.09	0.96	0.11	0.27	1.29	0.19
	0.85	0.25	0.84	0.11	0.96	0.13	0.30	1.20	0.24
	0.9	0.30	0.80	0.13	0.95	0.15	0.32	1.07	0.33
	0.95	0.43	0.68	0.21	0.92	0.22	0.29	0.86	0.57
	0.99	0.48	0.59	0.31	0.88	0.32	0.27	0.76	0.78
2000 WMH & 10000 non-WMH	0.8	0.45	0.67	0.18	0.87	0.21	0.22	0.88	0.60
	0.85	0.48	0.63	0.20	0.83	0.23	0.21	0.83	0.67
	0.9	0.52	0.58	0.23	0.78	0.26	0.18	0.79	0.75
	0.95	0.56	0.45	0.35	0.66	0.35	0.13	0.74	0.89
	0.99	0.54	0.37	0.47	0.59	0.44	0.14	0.78	0.95

SI: Dice similarity index; FDR: false-discovery ratio; FNR: false-negative ratio; FDRc: cluster-level FDR; FNRc: cluster-level FNR; DER: detection error rate; OER: outline error rate; ICC: interclass correlation coefficient

Supplementary Table 3: Third Round of BIANCA Settings Optimization
Model Starting Point: Modality = FLAIR + T2; Patch Size = 0; Spatial Weighting = 0; Training Point Location = any; Training Point Number = 2000 WMH & 10000 non-WMH; Threshold=0.95

BIANCA options		Overlap with manual mask						Volumetric correspondence	
Setting	Threshold	SI	FDR	FNR	FDRc	FNRc	DER	OER	ICC
Modality									
FLAIR	0.95	0.40	0.49	0.57	0.84	0.39	0.50	0.71	0.80
FLAIR+T2	0.95	0.51	0.41	0.45	0.77	0.32	0.36	0.62	0.86
Patch Size									
0	0.95	0.53	0.43	0.38	0.79	0.27	0.37	0.56	0.93
3	0.95	0.51	0.42	0.44	0.73	0.38	0.36	0.61	0.90
6	0.95	0.50	0.40	0.48	0.68	0.36	0.35	0.65	0.89
9	0.95	0.52	0.39	0.46	0.71	0.34	0.31	0.65	0.89
Spatial Weight									
0	0.95	0.51	0.41	0.45	0.77	0.32	0.36	0.62	0.86
1	0.95	0.58	0.41	0.35	0.71	0.33	0.14	0.70	0.91
10	0.95	0.43	0.52	0.56	0.73	0.50	0.21	0.93	0.84
5	0.95	0.51	0.47	0.44	0.70	0.44	0.16	0.81	0.94
Training Point Location									
any	0.95	0.51	0.41	0.45	0.77	0.32	0.36	0.62	0.86
noborder	0.95	0.52	0.43	0.41	0.79	0.29	0.38	0.58	0.82
surround	0.95	0.27	0.29	0.80	0.46	0.67	0.45	1.00	0.70
Training Point Number									
2000 WMH & 10000 non-WMH	0.8	0.47	0.60	0.23	0.89	0.18	0.56	0.50	0.47
	0.85	0.49	0.57	0.26	0.87	0.20	0.52	0.51	0.55
	0.9	0.51	0.52	0.30	0.84	0.23	0.47	0.51	0.66
	0.95	0.51	0.41	0.45	0.77	0.32	0.36	0.62	0.86
	0.99	0.44	0.35	0.60	0.69	0.40	0.32	0.79	0.90
10000 WMH & 50000 non-WMH	0.8	0.52	0.49	0.32	0.82	0.25	0.43	0.52	0.72
	0.85	0.53	0.45	0.35	0.80	0.28	0.39	0.54	0.78
	0.9	0.53	0.41	0.40	0.76	0.31	0.36	0.57	0.85
	0.95	0.48	0.33	0.56	0.66	0.41	0.31	0.73	0.90
	0.99	0.38	0.29	0.69	0.59	0.49	0.29	0.94	0.83
2000 WMH & 58000 non-WMH	0.8	0.53	0.40	0.42	0.75	0.33	0.35	0.59	0.88
	0.85	0.52	0.37	0.47	0.71	0.37	0.33	0.63	0.90
	0.9	0.49	0.33	0.54	0.67	0.41	0.31	0.70	0.92
	0.95	0.37	0.27	0.71	0.53	0.54	0.30	0.96	0.83
	0.99	0.25	0.25	0.83	0.45	0.65	0.39	1.10	0.60

SI: Dice similarity index; FDR: false-discovery ratio; FNR: false-negative ratio; FDRc: cluster-level FDR; FNRc: cluster-level FNR; DER: detection error rate; OER: outline error rate; ICC: interclass correlation coefficient

Supplementary Table 4: Fourth Round of BIANCA Settings Optimization
Model Starting Point: Modality = FLAIR + T2; Patch Size = 0; Spatial Weighting = 1; Training Point Location = any; Training Point Number = 2000 WMH & 58000 non-WMH; Threshold=0.8

BIANCA options		Overlap with manual mask						Volumetric correspondence	
Setting	Threshold	SI	FDR	FNR	FDRc	FNRc	DER	OER	ICC
Modality									
FLAIR	0.95	0.59	0.39	0.33	0.66	0.34	0.13	0.68	0.92
FLAIR+T2	0.95	0.61	0.39	0.32	0.65	0.31	0.12	0.67	0.92
Patch Size									
0	0.95	0.61	0.41	0.29	0.70	0.27	0.14	0.64	0.94
3	0.95	0.60	0.41	0.31	0.63	0.37	0.13	0.67	0.93
6	0.95	0.58	0.41	0.36	0.56	0.42	0.14	0.71	0.93
9	0.95	0.57	0.42	0.37	0.57	0.41	0.14	0.72	0.93
Spatial Weight									
0	0.95	0.53	0.40	0.42	0.75	0.34	0.36	0.58	0.87
1	0.95	0.61	0.40	0.31	0.65	0.31	0.12	0.67	0.92
10	0.95	0.47	0.48	0.51	0.67	0.51	0.18	0.87	0.85
5	0.95	0.54	0.45	0.41	0.64	0.42	0.15	0.77	0.95
Training Point Location									
any	0.95	0.61	0.40	0.31	0.65	0.31	0.12	0.67	0.92
noborder	0.95	0.60	0.44	0.27	0.67	0.27	0.12	0.68	0.88
surround	0.95	0.55	0.22	0.52	0.48	0.46	0.15	0.75	0.92
Training Point Number									
2000 WMH & 10000 non-WMH	0.7	0.42	0.70	0.13	0.91	0.14	0.21	0.95	0.52
	0.75	0.46	0.66	0.15	0.89	0.17	0.19	0.89	0.60
	0.8	0.48	0.64	0.16	0.88	0.18	0.18	0.87	0.63
	0.85	0.51	0.60	0.18	0.86	0.19	0.17	0.82	0.70
	0.9	0.54	0.55	0.21	0.84	0.23	0.15	0.77	0.78
	0.95	0.58	0.41	0.35	0.71	0.33	0.14	0.70	0.91
	0.99	0.54	0.33	0.49	0.63	0.41	0.14	0.78	0.94
10000 WMH & 50000 non-WMH	0.7	0.53	0.57	0.18	0.85	0.20	0.17	0.76	0.72
	0.75	0.57	0.52	0.21	0.81	0.22	0.15	0.72	0.78
	0.8	0.58	0.50	0.22	0.78	0.23	0.14	0.71	0.80
	0.85	0.59	0.46	0.25	0.74	0.26	0.13	0.68	0.85
	0.9	0.61	0.40	0.30	0.68	0.29	0.13	0.66	0.89
	0.95	0.57	0.28	0.46	0.54	0.40	0.13	0.72	0.93
	0.99	0.49	0.21	0.60	0.43	0.46	0.15	0.87	0.88
2000 WMH & 58000 non-WMH	0.7	0.59	0.47	0.25	0.74	0.27	0.13	0.69	0.86
	0.75	0.60	0.42	0.30	0.67	0.30	0.12	0.67	0.90
	0.8	0.61	0.40	0.31	0.65	0.31	0.12	0.67	0.92
	0.85	0.61	0.36	0.36	0.60	0.34	0.12	0.66	0.94
	0.9	0.59	0.30	0.43	0.53	0.38	0.13	0.69	0.95
	0.95	0.47	0.20	0.62	0.39	0.51	0.16	0.89	0.86
	0.99	0.34	0.14	0.76	0.32	0.60	0.23	1.09	0.66

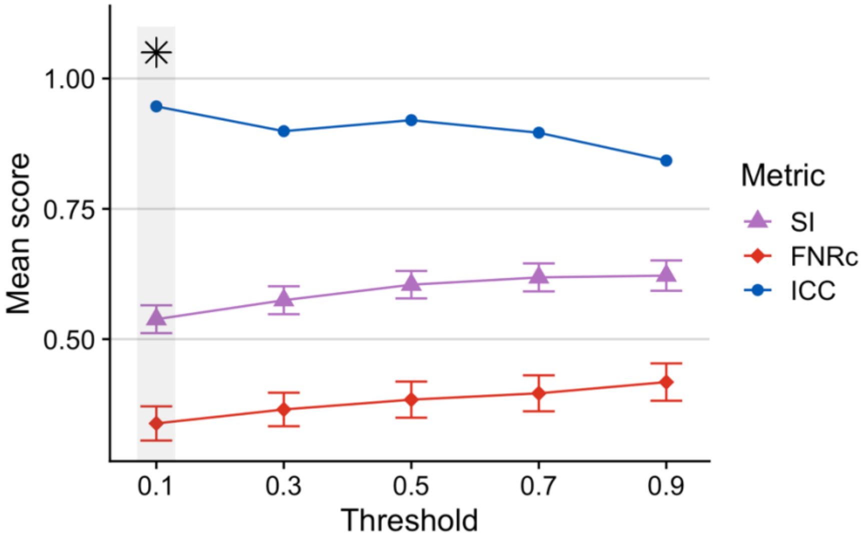

Supplementary Figure 1: Comparison of SAMSEG performance at different lesion probability thresholds. SAMSEG performed best with a 0.1 threshold, which is a lower threshold than the default settings (0.3 default). Better performance was indicated by higher interclass correlation coefficient (ICC) and lower cluster false-negative ratio (FNRc) scores, despite having lower Dice Similarity Index (SI). Grey bars and black asterisks indicate the best-performing setting.

Supplementary Table 5: SAMSEG thresholding performance scores

BIANCA options	Overlap with manual mask						Volumetric correspondence	
Threshold	SI	FDR	FNR	FDRc	FNRc	DER	OER	ICC
0.1	0.54	0.57	0.16	0.77	0.34	0.20	0.73	0.95
0.3	0.57	0.51	0.19	0.74	0.37	0.19	0.66	0.90
0.5	0.60	0.46	0.22	0.71	0.38	0.19	0.60	0.92
0.7	0.62	0.42	0.26	0.67	0.40	0.18	0.58	0.90
0.9	0.62	0.36	0.33	0.65	0.42	0.19	0.57	0.84

Note: Table presents performance scores for Dataset 1 (chronic stroke cohort) comparing performance between different lesion probability thresholds applied to SAMSEG segmentation (default setting is 0.3)
